# Micro RNA-155 inhibitor as a potential therapeutic strategy for the treatment of acute kidney injury (AKI): a nanomedicine perspective

**DOI:** 10.1039/c7ra13440a

**Published:** 2018-04-30

**Authors:** Shunjie Chen, Jianping Shan, Wei Niu, Fujun Lin, Shuang Liu, Ping Wu, Lijing Sun, Wei Lu, Gengru Jiang

**Affiliations:** Department of Nephrology, Xin Hua Hospital Affiliated to Shanghai JiaoTong University School of Medicine Shanghai 200092 P. R. China wb27lx@163.com gengrujiang@yahoo.com +86-13422121238 +86-13422121238

## Abstract

In this study, we have prepared miR-155 inhibitor-loaded liposome vesicles for the effective treatment of acute kidney injury. The efficacy of liposomal miR-155 inhibitor in the expression of miR-155, mortality in animals, the expression of TNF-α-IL6, and the expression of SOCS1–STAT1 were evaluated. The loading of miR-155 inhibitor into liposomes conferred the much needed colloidal stability and efficient delivery to the renal tissues. The study clearly shows that miR-I-LV significantly decreases the expression of miR-155 in kidneys compared to LPS. Administration of miR-I-LV remarkably reduced the pathological concerns of the kidneys with a marked decrease in inflammatory cell infiltration. Scrambled miR-155 did not have any effect on the expression of these markers; however miR-I-LV showed a remarkable ability to decrease the expression of TNF-α and IL-6 in kidney tissues indicating an ability to treat acute kidney infections. Overall, administration of miR-155 inhibitor effectively alleviated LPS-induced kidney injury by significantly suppressing TNF-α and IL-6 in kidney tissue and by remarkably increasing the expression of mRNA of SOCS1 and STAT1. The present results suggest that miR-155 inhibitor could be used in an effective targeting strategy for the treatment of acute kidney injury (AKI).

## Abstract 2

In this study, we have prepared miR-155 inhibitor-loaded liposome vesicles for the effective treatment of acute kidney injury.

## Graphical abstract

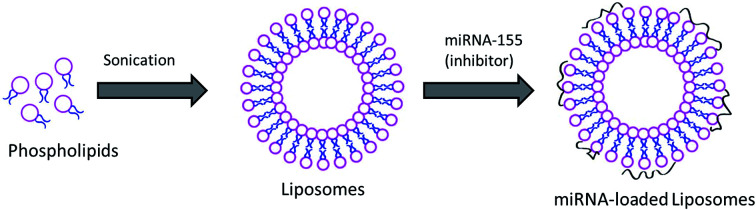

## Introduction

Acute kidney injury (AKI) is a disease which is defined by a number of clinical events that lead to the loss of regular functions of the kidney such as a reduction in glomerular filtration rate (GFR).^1^ The main causes of AKI are reported to be ischemia and toxins that affect the performance of tubulus and glomerulus functions. In particular, more than 50% of pediatric patients with sepsis have been reported to develop AKI.^2,3^ AKI is considered to be one of the risk factors responsible for the development of chronic kidney disease and other forms of renal diseases and non-renal diseases such as cardiovascular complications.^4,5^ Despite the tremendous progress in the knowledge and management of AKI, the incidence of AKI keeps increasing every year. Specifically, in pediatric patients with sepsis-associated AKI, the mortality rate is more than 60%. Mechanistically, AKI occurs due to nitric oxide pathway activation, followed by the adhesion of leukocytes, and reactive oxygen species (ROS) generally, and renal inflammation.^6^

Micro RNAs (miRNAs) are endogenous and non-coding RNA molecules with a short length of 18–25 nucleotides that target messenger RNA (gene regulation) and affect translational repression.^7,8^ miRNA is involved in the regulation of inflammatory responses and has functional roles in the pathogenesis of several diseases in vital organs. Recently studies have highlighted the importance of miR-155, miR-150, and miR-146 in the regulation of inflammatory responses.^9^ To be specific, miR-155 is actively involved in the initiation of inflammation in liver cells and is largely responsible for kidney damage by regulating the immune response.^10^ It has been reported that pretreatment with antagonist miR-155 reduces vascular leaks *via* the regulation of endothelial activation.^11^ The functional role of miR-155 in AKI was further demonstrated by the fact that lipopolysaccharide (LPS) induces the expression of miR-155 in damaged kidneys while it decreases the expression of SOCS1 in RAW264.7 cells.^12^ Furthermore, resveratrol has been reported to protect the inflammatory response in macrophages by suppressing miR-155 expression and downregulating signal transducers and activators of transcription (STAT)1/STAT3.^13,14^ The signaling components of inflammation (cytokines) such as TNF-α or IL6 are located in the STAT-binding sites and are therefore stimulated by miR-155-based signaling pathways.^15^ SOCS, a regulatory protein, is directly involved in cardioprotective effects by upregulating angiogenic factors. Interestingly, miR-155 expression inhibits the expression of the SOCS1 protein.^16^ Inhibition of miR-155 can directly increase the expression of SOCS1 and thereby suppress the gene expression of cytokines including IL6 or TNF-α and other inflammatory genes responsible for inflammation and kidney damage. It has been reported that inhibition of miR-155 protects several vital organs such as the liver, kidneys and lungs from injury.^17^

Overall, the main aim of the present study was to investigate the protective effect of the inhibition of miR-155 on acute kidney damage and study the influence of miR-155 on LPS induced inflammation or kidney damage. We hypothesized that inhibition of miR-155 expression by anti-miR-155 could downregulate the respective gene expression and induce the SOCS gene expression which might regulate the inflammatory response and thereby cure or alleviate the acute kidney damage. Towards this goal, we have designed a liposome as a novel carrier to encapsulate the anti-miR-155 and systemically administer it. Liposomes are one of the most investigated carrier systems for drug and gene delivery in the body. Many of the liposome based products are either on the market or in various phases of clinical trial.^18,19^ As such, miRNA would be unstable in a dynamic systemic environment whereas miRNA-loaded liposomes are expected to add colloidal stability and increase delivery to the target site. The underlying mechanism involved in the inflammatory response and regulatory pathways are studied in detail in the present study.

## Results and discussion

**Fig. 1:**
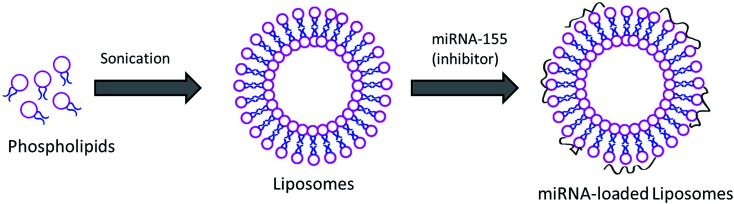
Schematic representation of the preparation of miRNA-155 inhibitor loaded liposomal vesicles.

Acute kidney infection is one of the serious problems in the body and kidneys are affected badly during AKI. Although mortality due to AKI has fallen over the past decade, it is an important cause of death in critical patients. In this regard, increasing evidence suggests that certain genes play an important role in the pathology and treatment of specific diseases. It has been reported that miRNA has an active role in AKI progression. miR-155 belongs to a miRNA family which gets involved in multiple-factor induced kidney inflammation. It has been reported that alterations in the miR-155 expression level in kidneys have a direct relationship with the seriousness of AKI in the human body. The functional role of miR-155 in AKI was further demonstrated by the fact that lipopolysaccharide (LPS) induces the expression of miR-155 in damaged kidneys while it decreases the expression of SOCS1 in RAW264.7 cells. It has been reported that pretreatment with antagonist miR-155 reduced vascular leaks *via* the regulation of endothelial activation. However, systemic administration of a lipofectamine complex or miRNA will not be stable and its clinical benefit will be compromised.^20^ Therefore, in the present study, we have designed a liposome nanocarrier system to load and administer to the systemic environment. Liposomes are one of the highly studied nanocarrier systems in clinical trials and are reported to be stable under harsh systemic conditions (Fig. 1).

### Physicochemical characterization of miRNA-loaded liposomes

**Fig. 2:**
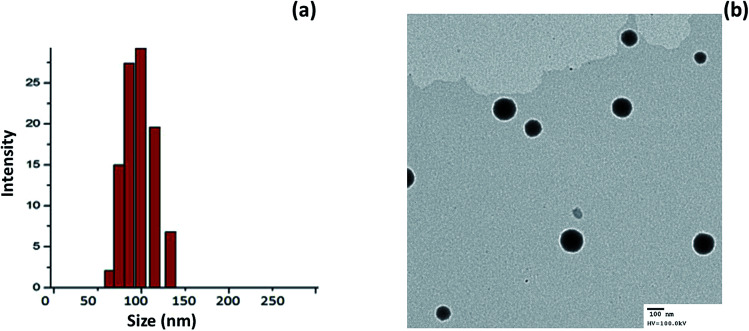
(a) Particle size analysis of miR-I-LV using a dynamic light scattering (DLS) method; (b) transmission electron microscope (TEM) image of miR-I-LV.

Cationic liposomes were prepared using thin-film hydration technology. We have used DDAB in order to impart a positive charge to the surface of the liposomes. The average particle size of miR-I-LV was observed to be ∼120 nm with an excellent dispersity index (PDI) of ∼ 0.12 (Fig. 2a). The surface charge of the liposomes before and after conjugation with miRNA was observed to be 26.45 ± 1.25 mV and 21.56 ± 1.45 mV, respectively. A slight decrease in the surface charge was attributed to the physical conjugation of miRNA to the liposomes. The interaction of the negative charge of the miRNA and the positive charges of the liposomes formed stable nanoparticles. Moreover, nanosized particles will accumulate in tissue and avoid immediate clearance from blood circulation. The morphology of the particles was evaluated using a transmission electron microscope (TEM). As seen, particles were spherically shaped and uniformly spread on the TEM grid. Due to the low molecular weight of miRNA, surface conjugation of miRNA was not observed in the TEM image (Fig. 2b).

### Gel retardation assay

**Fig. 3:**
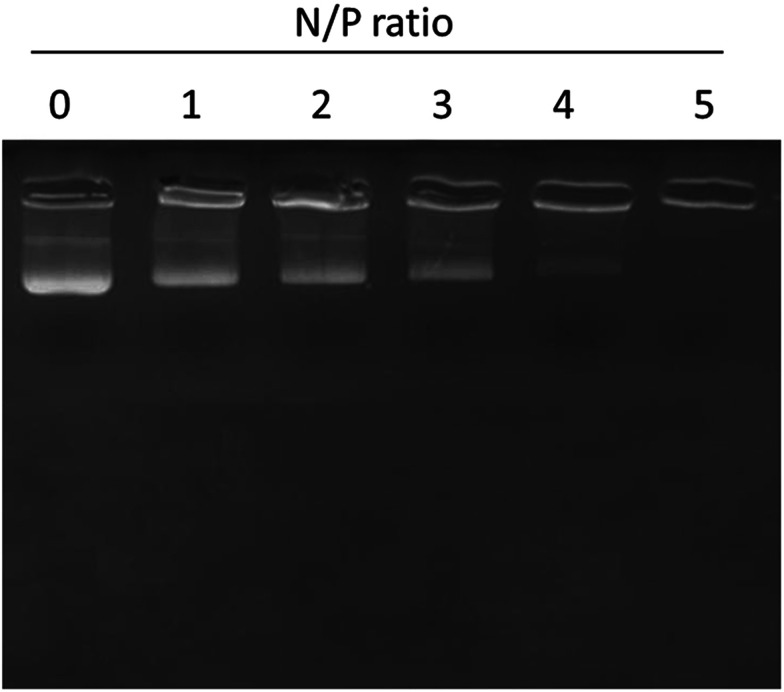
Gel retardation analysis of miR-155 inhibitor using gel electrophoresis. N/P ratios of miR-155 and liposome nanoparticles were changed and the binding patterns were evaluated.

The surface conjugation or conjugation efficiency of miRNA on the liposomes was proven by gel retardation assay. As seen, 100% conjugation was observed with the increase in the N/P ratio (Fig. 3). In particular, 100% conjugation was observed at a N/P ratio of 5 where migration of miRNA was completely retarded due to the high conjugation efficiency of the liposomes towards miRNA.

### miR-155 inhibitor controls the expression of miR-155 in kidneys

**Fig. 4:**
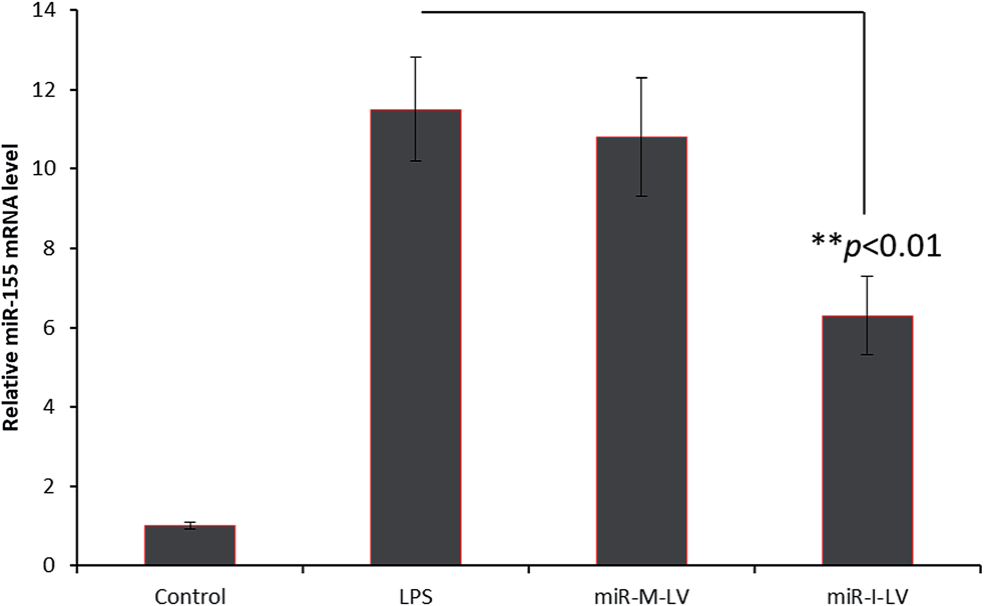
miR-155 expression in renal tissues after the administration of LPS, miR-M-LV and miR-I-LV, respectively.

In the present study, we have developed an endoxemia model to study the effect of miR-I-155 inhibitor on AKI. Throughout the study, we have used LPS at a standard dose of 20 mg kg^−1^. To study the effect of miR-155 inhibitor, mice were administered with LPS, miR-M-LV and miR-I-LV *via* the tail (2 times a day) for 3 days (Fig. 4). The kidneys were harvested from the animals and subjected to respective study. As seen, LPS showed an 8-fold higher expression of miRNA-155 in the kidneys compared to that of the non-treated control indicating that the inflammation of the kidneys (caused by LPS administration) results in the higher expression of miR-155. miR-I-LV significantly decreased the expression of miR-155 in the kidneys compared to LPS whereas scrambled miRNA-155 has no effect on the expression of miR-155.

### Effect of miR-155 inhibitor on the mortality rate of mice

**Fig. 5:**
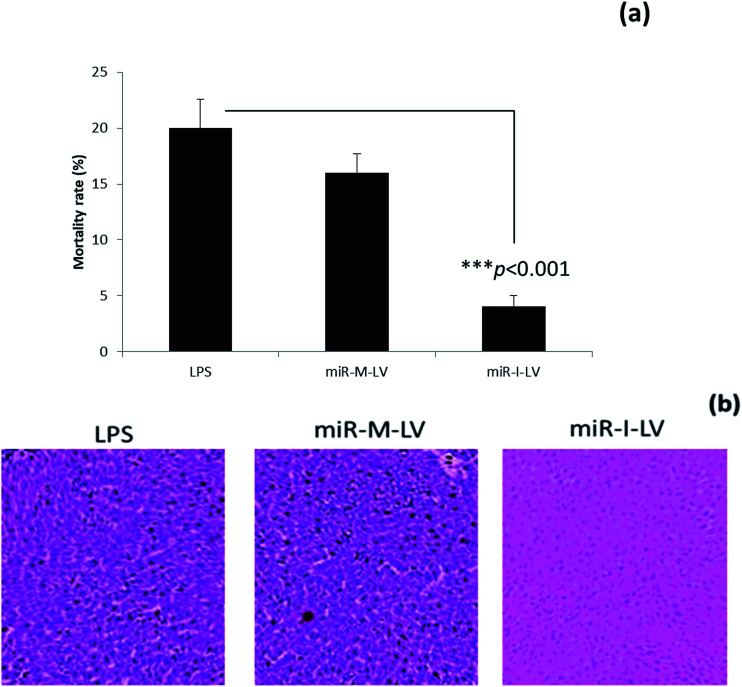
Effect of miR-155 inhibitor on the kidney injury of mice; (a) mortality rate of mice after the administration of respective formulations; (b) histology analysis of kidneys from the respective groups of mice after 12 h of administration.

The mortality rate of mice administered with different formulations was calculated at the end of a 12 h study period. As seen, LPS treated mice showed a high mortality rate of 20% while scrambled miRNA also killed mice at a similar rate. Notably, miR-I-LV treated mice showed a remarkably lower mortality rate (Fig. 5a). Visual inspection indicated that LPS treated mice developed serious symptoms whereas miR-I-LV treated mice were healthy without any side effects. Kidney sections were paraffinized to perform histology analysis (Fig. 5b). As expected, the LPS treated group showed remarkable pathological symptoms such as inflammatory cell infiltration, neutrophil infiltration in the glomerulus, tubular epithelial swelling, necrosis of the glomerulus and degeneration of renal tubules. The administration of miR-I-LV remarkably reduced the pathological concerns of the kidneys with a marked decrease in inflammatory cell infiltration. These results show the potential of miR-I-LV (miR-155 inhibitor) in managing the symptoms of AKI.

### Effect of miR-I-LV on TNF-α and IL-6 expression in kidneys

**Fig. 6:**
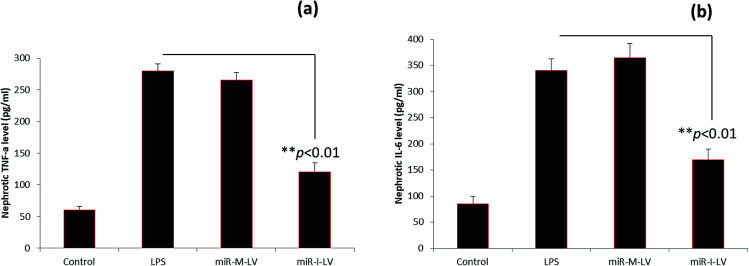
Effect of miR-155 inhibitor on the expression of inflammatory signals; (a) inhibitory ability of miR-I-LV on the expression of TNF-α; (b) inhibitory ability of miR-I-LV on the expression of IL-6.

The role of miR-I-LV in the expression of TNF-α and IL-6 in kidney tissue is evaluated. As seen, LPS administration significantly increases the expression of TNF-α and IL-6 in renal tissues compared to the blank control (Fig. 6a and b). The scrambled miR-155 did not have any effect on the expression of these markers; however miR-I-LV showed a remarkable ability to decrease the expression of TNF-α and IL-6 in kidney tissues indicating an ability to treat acute kidney infections. Acute kidney injury is likely to progress by the infiltration of proinflammatory mediums such as TNF-α and IL-6 which are activated by NF-Kb based transcription and gene regulation. The ability of miR-I-LV to significantly decrease the expression of TNF-α and IL-6 explains its ability to decrease inflammation at the diseased site. The inhibition of miR-155 by miR-I-LV could potentially block the inflammatory response and could most likely inhibit caspase cascade. There are several incidents wherein regulation of TNF-α and IL-6 resulted in a better therapeutic effect. For example, astragaloside IV has been reported to suppress the LPS-mediated increase of TNF-α and IL-6 in endotoxinemia models. Similarly, eupafolin regulated anti-inflammatory properties and proved to be beneficial for AKI treatment.^21,22^ These results clearly indicate that control of inflammatory mediums is absolutely important.

### Effect of miR-I-LV on SOCS1 and STAT1 expression in kidneys

**Fig. 7:**
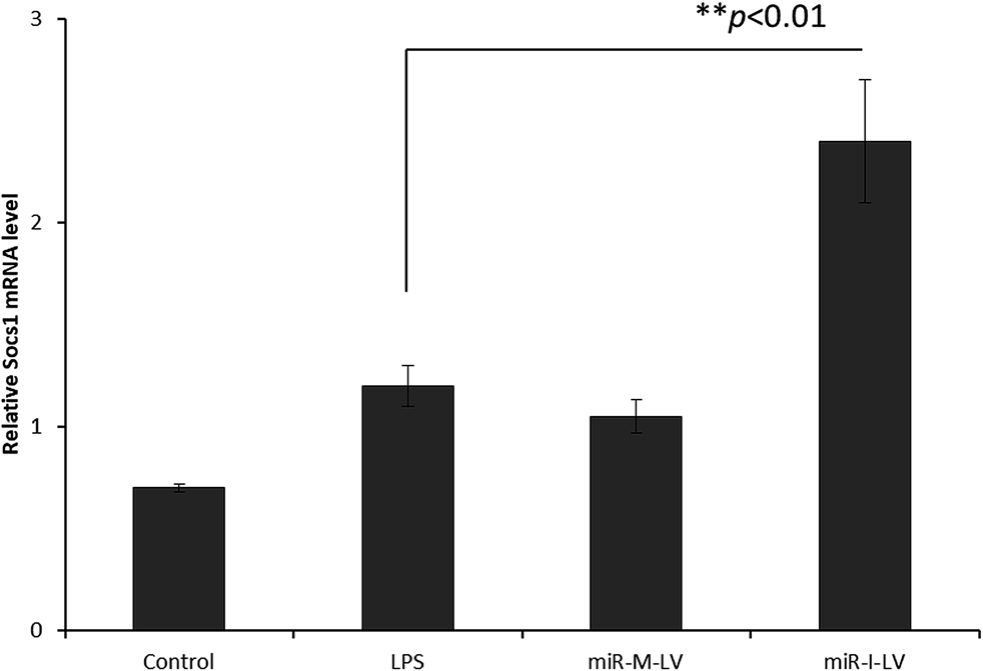
Effect of miR-155 inhibitor on the expression of target genes; PCR analysis of the expression of mRNA of SOCS1 after the administration of LPS, miR-M-LV and miR-I-LV, respectively.

**Fig. 8:**
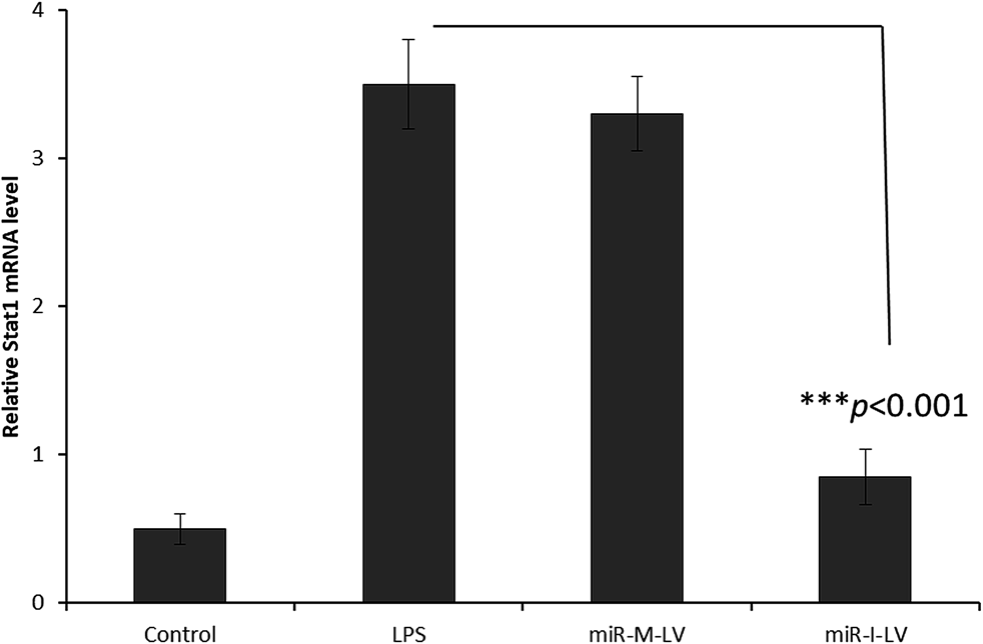
Effect of miR-155 inhibitor on the expression of target genes; PCR analysis of the expression of mRNA of STAT1 after the administration of LPS, miR-M-LV and miR-I-LV, respectively.

It has been reported that miR-155 targets negative signaling pathways of JAK/STAT1 by targeting SOCS1; therefore miR-155 inhibitor’s effect on these signaling pathway components has been evaluated in mice. As seen (Fig. 7), miR-155 inhibitor has directly increased the expression of mRNA of SOCS1 in renal tissue indicating its ability to control the negative signaling pathways. LPS administration controlled the expression of mRNA of SOCS1. Consistently, STAT1 expression has been controlled after the administration of miR-155 while no significant difference was observed between LPS and miR-155 inhibitor in the STAT1 expression (Fig. 8). There are several reports which suggest that the JAK/STAT pathway is actively involved in organ damage like AKI. To be specific, the JAK/STAT pathway is the potential target of inflammation and miR-155 expression. In the nucleus, STATs transcribe the target genes and promote the release of mediums such as STAT1 or STAT3. In this study, we have clearly observed the increased expression of SOCS1 mRNA after the administration of miR-155 inhibitor resulting in the negative regulation of STAT1, JAK2 or STAT3 expression.^23–25^

This study revealed that miR-155 is associated with inflammation by the regulation of SOCS1–STAT pathways. And since SOCS1 is the target point of miR-155, miR-I-LV-based AKI treatment relies on the regulation of the aforementioned signaling pathways. Overall, administration of miR-155 inhibitor effectively alleviated LPS-induced kidney injury by significantly suppressing TNF-α and IL-6 in kidney tissue and by remarkably increasing the expression of mRNA of SOCS1 and STAT1. All these results point towards the fact that miR-155 loaded in a liposomal nanocarrier could effectively tackle kidney injury by altering the signaling pathways.

## Materials and methods

miR-155 (5′-ACCCCUAUCACAAUUAGCAUUAA-3) and anti-miR-155 (5′-CAGUACUUUUGUGUAGUACAA-3′) were synthesized by Genepharma (China). Lipopolysaccharide (LPS) was purchased from Sigma-Aldrich, China. Dimethyl-di-octadecyl-346 ammonium (DDAB), 1,2-distearoyl-*sn*-347 glycero-3-phosphoethanolamine-*N*-[amino(polyethylene glycol)-2000 (DSPE-PEG), and cholesterol were purchased from Avanti Polar Lipids.

### Preparation of miRNA-155 loaded liposomal formulations

miR-155 loaded liposome was prepared using a thin-film hydration method. Briefly, DDAB, cholesterol and DSPE-PEG at a molar ratio of 1 : 4 : 5 were dissolved in 1 ml of organic solvent and placed under an argon atmosphere for 30 min. The film was then transferred to a vacuum for 60 min to remove traces of organic solvent in the lipid film. The film was hydrated using 0.1× phosphate buffered saline (pH 7.4) and mechanically shaken for 60 min. The opaque solution was subjected to extrusion using a mini-extruder for 21 cycles with a filter size of 100 nm. The as-formed liposomes were loaded with miRNA using an incubation method. A 10 μg equivalent of miRNA was incubated with cationic liposomes (1 ml) and incubated for 12 h at 4 °C. The formulation was centrifuged to remove the unloaded miRNA and stored in a refrigerator until further processing.

### Characterization of liposomes

The particle size of the liposomes was evaluated using a Zetasizer NANO ZS (Malvern Instruments, UK), using the principle of dynamic light scattering (DLS). The particles were diluted properly and the measurements were performed at 37 °C in triplicate. The morphology of the particles was evaluated using transmission electron microscopy (TEM) using a JEM-2000EX (JEOL, Japan). The nanoparticles were stained with phosphotungstic acid and allowed to rest for 15 min. The particles were then drained of water and dried under an infrared light for 5 min. The particles were imaged using TEM.

### *In vivo* animal model and efficacy analysis

All animal procedures were performed in accordance with the Guidelines for Care and Use of Laboratory Animals of Shanghai Jiao Tong University School of Medicine and approved by the Institutional Animal Ethical Committee (IAEC) of Shanghai Jiao Tong University. The mice were equally divided between different groups including blank, LPS, mutant control and miR-I-LV, respectively. To all the respective groups, LPS was administered. The mice were anesthetized using pentobarbital, and the blank group was administered with saline and the LPS group was administered with LPS at a dose of 20 mg kg^−1^. The efficacy of miR-155 was evaluated in LPS-induced kidney injured mice by injecting miR-155 inhibitor (miR-I-LV) into the mutant group. The last two groups of mice were injected with LPS at a fixed dose of 20 mg kg^−1^. All the mice were sacrificed 12 h after the administration of therapeutic agents. The kidneys were collected and frozen and one piece was subjected to reverse transcription-quantitative polymerase chain reaction (RT-qPCR) analysis and another piece was subjected to H&E staining.

### ELISA assay

The amount of biomarker (TNF-α and IL-6) in the kidneys was evaluated as per the standard protocol set by the manufacturers kit (ELISA Kit). The amount of protein was estimated using a BCA bicinchoninic acid protein assay kit. The amounts of TNF-α and IL-6 were presented per milligram of protein.

### H&E analysis of kidney sections

The mice were sacrificed and the kidneys were surgically removed. The kidney sections were fixed with 4% paraformaldehyde and incubated overnight to fix all the cells. The next day, the kidneys were transferred to 75% ethanol and embedded in paraffin. The paraffin-embedded samples were cut into tiny samples of 5 μm thickness. The cells were then stained with hematoxylin and eosin (H&E) and established protocols were followed to complete the whole process. The sections were observed under a fluorescence microscope (Olympus, Tokyo, Japan).

### Reverse transcription-quantitative polymerase chain reaction (RT-qPCR)

The parts of the kidneys which were kept in RNAse Later were used for PCR analysis. First, the tissue sample was broken using a probe sonicator and total RNA was collected using TRIZOL. The established protocols were followed to purify the RNA from the bulk samples and the amount of total RNA was quantified using NANODROP equipment. Thereafter, 0.5 μg total RNA was reverse-transcribed using a Thermo Scientific Revert Aid First Strand cDNA synthesis kit. RT-PCR analysis for miR-155 inhibitor was performed using a SYBR Green PCR Master Mix (Takara, Shiga, Japan) on the Step one plus system (Thermo Fisher Scientific). The PCR conditions were set at 95 °C for 3 minutes, 40 cycles of 95 °C for 12 seconds and 62 °C for 40 seconds. GADPH mRNA was set as an appropriate control to normalize all the data.

### Statistical analysis

Statistical significance was analyzed using a *t*-test. The resultant *P*-value was expressed as *P < 0.05 and **P < 0.01. Differences were considered to be statistically significant if the *P*-value was <0.05. All the results are expressed as mean ± S.D and experiments were performed in triplicate unless otherwise mentioned.

## Conclusions

In this study, we have prepared miR-155 inhibitor-loaded liposome vesicles for the effective treatment of acute kidney injury. The loading of miR-155 inhibitor in liposomes conferred the much needed colloidal stability and efficient delivery to renal tissues. The study clearly showed that miR-I-LV significantly decreased the expression of miR-155 in kidneys compared to LPS. The administration of miR-I-LV remarkably reduced the pathological concerns of kidneys with a marked decrease in inflammatory cell infiltration. The scrambled miR-155 did not have any effect on the expression of these markers; however miR-I-LV showed a remarkable ability to decrease the expression of TNF-α and IL-6 in kidney tissues indicating an ability to treat acute kidney infections. Overall, administration of miR-155 inhibitor effectively alleviated LPS-induced kidney injury by significantly suppressing TNF-α and IL-6 in kidney tissue and by remarkably increasing the expression of mRNA of SOCS1 and STAT1. The present results suggest that miR-155 inhibitor could be an effective targeting strategy for the treatment of acute kidney injury (AKI).

## Conflicts of interest

There are no conflicts to declare.

## Supplementary Material
